# Characteristics of children readmitted with severe pneumonia in Kenyan hospitals

**DOI:** 10.1186/s12889-024-18651-2

**Published:** 2024-05-16

**Authors:** Diana Marangu-Boore, Paul Mwaniki, Lynda Isaaka, Teresiah Njoroge, Livingstone Mumelo, Dennis Kimego, Achieng Adem, Elizabeth Jowi, Angeline Ithondeka, Conrad Wanyama, Ambrose Agweyu

**Affiliations:** 1 Paediatric Pulmonology Division, Department of Paediatrics and Child Health, University of Nairobi, Nairobi, Kenya; 2 Epidemiology and Demography Department, KEMRI-Wellcome Trust Research Programme, Nairobi, Kenya; 3 Department of Health, Kisumu County, Kenya; 4 Department of Health, Nairobi County, Kenya; 5 Department of Health, Nakuru County, Kenya; 6 Department of Infectious Disease Epidemiology, London School of Hygiene and Tropical Medicine, London, Great Britain

**Keywords:** Lower respiratory tract infections, Paediatrics, Adolescents, Low-and-middle-income countries, Africa, Recurrent pneumonia, Persistent pneumonia

## Abstract

**Background:**

Pneumonia is a leading cause of childhood morbidity and mortality. Hospital re-admission may signify missed opportunities for care or undiagnosed comorbidities.

**Methods:**

We conducted a retrospective cohort study including children aged $\ge$2 months-14 years hospitalised with severe pneumonia between 2013 and 2021 in a network of 20 primary referral hospitals in Kenya. Severe pneumonia was defined using the 2013 World Health Organization criteria, and re-admission was based on clinical documentation from individual patient case notes. We estimated the prevalence of re-admission, described clinical management practices, and modelled risk factors for re-admission and inpatient mortality.

**Results:**

Among 20,603 children diagnosed with severe pneumonia, 2,274 (11.0%, 95% CI 10.6–11.5) were readmitted. Re-admission was independently associated with age (12–59 months vs. 2–11 months: adjusted odds ratio (aOR) 1.70, 1.54–1.87; >5 years vs. 2–11 months: aOR 1.85, 1.55–2.22), malnutrition (weight-for-age-z-score (WAZ) <-3SD vs. WAZ> -2SD: aOR 2.05, 1.84–2.29); WAZ − 2 to -3 SD vs. WAZ> -2SD: aOR 1.37, 1.20–1.57), wheeze (aOR 1.17, 1.03–1.33) and presence of a concurrent neurological disorder (aOR 4.42, 1.70-11.48). Chest radiography was ordered more frequently among those readmitted (540/2,274 [23.7%] vs. 3,102/18,329 [16.9%], *p* < 0.001). Readmitted patients more frequently received second-line antibiotics (808/2,256 [35.8%] vs. 5,538/18,173 [30.5%], *p* < 0.001), TB medication (69/2,256 [3.1%] vs. 298/18,173 [1.6%], *p* < 0.001), salbutamol (530/2,256 [23.5%] vs. 3,707/18,173 [20.4%], *p* = 0.003), and prednisolone (157/2,256 [7.0%] vs. 764/18,173 [4.2%], *p* < 0.001). Inpatient mortality was 2,354/18,329 (12.8%) among children admitted with a first episode of severe pneumonia and 269/2,274 (11.8%) among those who were readmitted (adjusted hazard ratio (aHR) 0.93, 95% CI 0.82–1.07). Age (12–59 months vs. 2–11 months: aHR 0.62, 0.57–0.67), male sex (aHR 0.81, 0.75–0.88), malnutrition (WAZ <-3SD vs. WAZ >-2SD: aHR 1.87, 1.71–2.05); WAZ − 2 to -3 SD vs. WAZ >-2SD: aHR 1.46, 1.31–1.63), complete vaccination (aHR 0.74, 0.60–0.91), wheeze (aHR 0.87, 0.78–0.98) and anaemia (aHR 2.14, 1.89–2.43) were independently associated with mortality.

**Conclusions:**

Children readmitted with severe pneumonia account for a substantial proportion of pneumonia hospitalisations and deaths. Further research is required to develop evidence-based approaches to screening, case management, and follow-up of children with severe pneumonia, prioritising those with underlying risk factors for readmission and mortality.

**Supplementary Information:**

The online version contains supplementary material available at 10.1186/s12889-024-18651-2.

## Background

In many low-and-middle-income-countries (LMICs), children and adolescents disproportionately face the double burden of acute respiratory infectious diseases and chronic comorbidities, resulting in long-term impairment of lung function [1, 2], reduced quality of life [3], and mortality [4]. Presence of chronic comorbidities and prior severe pneumonia episodes increase the risk of pneumonia re-admission and is associated with substantial costs [5, 6]. Almost 10% of children with community acquired pneumonia have recurrent pneumonia [7, 8]. Recurrent pneumonia is defined as at least two episodes of pneumonia in one year or three episodes ever, with interval radiographic clearing of opacities; whereas persistent pneumonia is characterized by persistence of symptoms and radiographic abnormalities for more than one month [9]. Recurrent and persistent pneumonia have varied aetiologies that are known antecedents to bronchiectasis or childhood interstitial lung disease [8, 10, 11], including rare genetic conditions that are increasingly being reported in sub Saharan Africa [12–14]. Obstructive airway disease in children hospitalised with severe pneumonia may be underdiagnosed, and other differential diagnoses may not be considered [15, 16]. While predictors of repeated acute hospitalisation for paediatric asthma which tends to co-exist with pneumonia are known and relatively modifiable, those for severe pneumonia readmission in sub-Saharan Africa are lacking [17].

The World Health Organization (WHO) guidelines for pneumonia used in many LMICs comprise a syndromic approach to disease classification and management to enable health workers at the first level referral hospitals to provide basic care for young children [18]. However, the validity of the syndromic approach to pneumonia classification and management has yet to be tested among children presenting for severe illness after a previous episode of hospitalisation. Robust data on inpatient management of children with repeated hospitalisation for severe pneumonia are needed to guide appropriate policy recommendations on preventive, diagnostic, treatment, and follow-up measures to optimize care and outcomes.

The aims of this study were to: (i) establish the proportion of children aged 2 months to 14 years who were re-admitted with severe pneumonia in an established network of Kenyan public hospitals; (ii) determine the factors associated with severe pneumonia re-admissions in this population; (iii) describe the investigations and treatment provided to children re-admitted with severe pneumonia; and (iv) explore the risk factors associated with mortality among these children.

## Methods

### Reporting

The reporting of this observational study follows the Strengthening of Reporting of Observational studies in Epidemiology (STROBE) statement [19].

### Ethics, consent, and permissions

The Kenya Medical Research Institute (KEMRI) Scientific and Ethics Review Unit approved the collection of the deidentified data analysed in this study and waived the requirement for individual informed consent from patients whose data were used for this research. The Clinical Information Network (CIN) is run in collaboration with the Kenya Paediatric Association, Ministry of Health, and participating hospitals with the aim to improving routine clinical documentation for quality improvement, and for observational and interventional research [20].

### Study design and setting

We undertook a retrospective cohort study involving patients admitted to CIN hospitals. The CIN was initiated in October 2013 and comprised 20 purposefully selected county hospitals distributed across the densely populated central and western regions of Kenya during the period of this analysis [21, 22]. Patients are admitted by a junior clinician under the supervision of one or more hospital paediatricians. CIN implements the use of structured clinical forms for all admitted children, capturing biodemographic data, caregiver-reported medical history, including whether the patient was readmitted and vaccination status according to the national routine schedule, clinical signs, diagnoses, diagnostic tests ordered, treatment plan, and inpatient outcome. Decisions on clinical and nursing management are made at the discretion of hospital staff with access to basic laboratory and radiological services. Case management is based on the Kenya national paediatric guidelines [23] adapted from WHO guidelines [18] as a reference (Table 1). Patients are reviewed at least once daily for clinical progress. At hospital discharge, caregivers receive instructions on correct administration of continuing treatments and a follow up outpatient clinic visit is scheduled typically within two weeks.

**Table 1:** WHO pneumonia classification for children aged 2–59 months with cough and/or difficulty in breathing

No Pneumonia	Cough or cold
Pneumonia	Lower chest indrawing AND/OR Fast breathing - Respiratory rate ≥ 50 min (age 2–11 months) - Respiratory rate ≥ 40 min (age 12–59 months)
Severe Pneumonia	Pneumonia AND at least one danger sign - Oxygen saturations < 90% - Central cyanosis - Inability to drink or breastfeed - Only responsive to voice or pain or unconscious (not alert) - Grunting

### Study participants, data sources and variables

The study population comprised all children admitted to paediatric wards in CIN hospitals. Children hospitalised with a documented diagnosis of pneumonia were included. Children aged less than 2 months or more than 14 years, and those without severe pneumonia defined using the World Health Organization criteria between 2013 and 2021 were excluded. Trained data clerks extract deidentified individual patient data from routine hospital records into a REDCap database upon discharge [22, 24]. For this analysis, data retrieved comprised: the patient’s unique identifier, age, sex, hospital unique identifier, prior pneumonia admission (we could not compute the time from the first admission given the nature of the data), known comorbidities or risk factors such as malnutrition, anaemia clinically assessed as pallor, chronic neurological disorder, asthma, cardiac disease, renal disease, HIV, TB and sickle cell disease. We also extracted clinical data comprising management instituted during hospitalisation such as routine laboratory investigations e.g. full blood count; plain chest radiography; supportive treatment e.g. oxygen supplementation; antimicrobials treatment (first line, second line, third line antibiotics, coverage for atypical pathogens, antitubercular and antifungal medications); inhaled or systemic corticosteroids; and outcomes including discharge, referral or death. It should be noted that the structured forms used in CIN to document diagnostics and treatments only record affirmative entries (i.e., “Yes,” “Ordered,” or “Prescribed”). As a result, we inferred that entries which were not recorded on the forms were negative.

#### Statistical analysis

Data were exported to STATA (StataCorp. 2019. *Stata Statistical Software: Release 16*.1 College Station, TX: StataCorp LLC.) for statistical analysis. Descriptive statistics were summarized as frequencies with percentages for categorical data, means with standard deviations for continuous data that were normal and medians with interquartile range for continuous data that were skewed. We estimated the proportion of severe pneumonia re-admissions among children aged 2 months to 14 years hospitalised within the CIN.

A multivariable mixed-effects logistic model was fitted to determine factors associated with re-admission among patients with severe pneumonia in this clinical cohort and we reported adjusted odds ratios. CIN data are hierarchical because admission episodes are nested within 20 hospitals, thus we used a random intercept logistic regression model to account for clustering [25]. In the formula that follows, Y_ij_ denotes pneumonia readmission for the i^th^ child admitted in the j^th^ hospital (Y_*ij*_ = 1 denotes pneumonia readmission, while Y_*ij*_ = 0 denotes no pneumonia readmission); X_*1ij*_, through X_*kij*_ denote the k explanatory variables measured on the i^th^ child admitted in the j^th^ hospital (e.g., child’s age); and *α*_0*j*_ denotes the unobserved cluster effect. Our model had no hospital level variables. It incorporates cluster-specific random effects to account for the within‐cluster correlation of patient outcomes.

$$\mathrm{logit}(\Pr({\mathrm Y}_{ij}\;=\;1))\;=\;{\mathrm\alpha}_0\:+\:{\mathrm\alpha}_{\mathrm{oj}}\:+\:{\mathrm\alpha}_1X_{1ij}\;+\;\cdots\;+\;{\mathrm\alpha}_{\mathrm k}X_{kij}\;\mathrm{where}\;{\mathrm\alpha}_{0\mathrm j}\sim\mathrm N(0,\;T^2)$$

The assumption is made that the random effects are independent of the model covariates (X). Explanatory variables included age, sex, weight-for-age z- score (WAZ), vaccination status, previous wheeze and comorbidities including anaemia, HIV, TB, and neurological disorders. The Stata command “or: xtmelogit” was used to fit the random intercept model.

To determine factors associated with mortality among children with severe pneumonia readmissions, we fitted a multivariable mixed-effects Cox proportional hazard model (shared gamma frailty model) and reported adjusted hazard ratios with corresponding 95% confidence intervals. In the formula that follows, αj denotes the random effect associated with the j-th hospital. The shared frailty term has a multiplicative effect on the baseline hazard function: hi (t ) = h0(t ) exp(αj ) exp(Xj β) [26]. Explanatory variables included age, sex, weight-for-age z- score (WAZ), vaccination status, previous wheeze and comorbidities including anaemia, HIV, TB, and neurological disorders. We generated Cox proportional hazard regression plots for survival by type of pneumonia admission (first episode pneumonia and pneumonia re-hospitalisation); by age group (under 12 months, 12–59 months and over 5’s), gender, WAZ category (<-3 SD, -2 to -3 SD, >-2SD), vaccination and anaemia status. The Stata command “stcox” was used to fit the shared frailty Cox proportional hazard model. Censoring occurred at the time of death, discharge or referral, whichever came first.

For all models, explanatory variables were selected *a priori* based on existing literature, clinical relevance regarding effect on outcome, ease of identification for risk stratification, and biological plausibility. *P*-values were rounded to three decimal places and were deemed statistically significant if they were < 0.05 (2-sided). To handle missing data, we performed multiple imputation by chained equations generating 10 imputed datasets under the assumption that data were missing at random (MAR) [27].

## Results

**Fig. 1:**
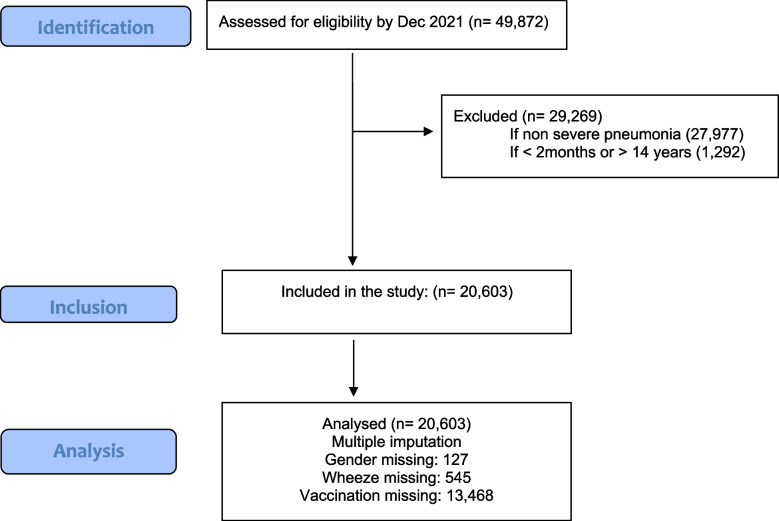
STROBE Flow Chart. Flow diagram of eligible study participants

 Out of 49,872 children admitted with pneumonia during the study period, 29,269 (59%) were excluded from the analysis. Exclusions were 27,977 children who did not have severe pneumonia, and 1,292 children aged < 2 months or > 14 years. Figure 1 shows the study population inclusion process. The main variables with missing values were gender, which was missing for 127 children (0.6%), wheeze which was missing for 545 children (2.7%), and vaccination status which was missing for 13,468 children (65%).

### Proportion of children re-admitted with severe pneumonia

Of the population included in the analysis comprising 20,603 children aged 2 months to 14 years hospitalised with severe pneumonia over the study period, 2,274 were readmitted, giving an estimated prevalence of 11.0% (95% CI 10.6–11.5) for readmissions among children with severe pneumonia.

### Characteristics of study participants

The median age of the study population was 12 months (IQR 6.0–24.0), 9,907/20,603 (48%) of children were under 12 months of age, 9,287/20,603 (45%) of the children were aged 12–59 months and 1,409/20,603 (6.8%) were aged 5–14 years. The majority of children were reported to be up to date with their vaccination status 6,950/7,135 (97%). However, most were severely or moderately underweight 17,018/20,603 (83%). Compared to children admitted for the first time with severe pneumonia, children re-admitted with severe pneumonia were slightly older (median age 14.4 months vs. 12 months), severely or moderately underweight (73.1% vs. 83.8%), were diagnosed with TB (1.8% vs. 0.7%), and neurological disorders comprising cerebral palsy, convulsive disorder or epilepsy (0.4% vs. 0.1%) more frequently (Table 2).

**Table 2:** Admission characteristics of study participants

Indicator	Levels	Total	Severe Pneumonia 1st Admission	Severe Pneumonia Readmission	*p*-value
		20,603	18,329	2,274	
Age months Median (IQR)		12.0 (6.0–24.0)	12.0 (6.0–24.0)	14.4 (9.6–28.8)	< 0.001
	Missing	0	0	0	
(%)	< 12mo	9,907 (48.1)	9,078 (49.5)	829 (36.5)	< 0.001
	12-59mo	9,287 (45.1)	8,030 (43.8)	1,257 (55.3)	
	Over 5yrs	1,409 (6.8)	1,221 (6.7)	188 (8.3)	
	Missing	0	0	0	
Gender (%)	Male	11,334 (55.4)	10,049 (55.2)	1,285 (56.8)	0.13
	Missing	127 (0.62)	114 (0.63)	13 (0.57)	
WAZ Median (IQR)		-1.1(-2.46, -0.46)	-1.1(-2.40, -0.03)	-1.7(-3.25, -0.51)	< 0.001
	Missing	812	718	94	
(%)	<-3SD	14,225 (69.0)	12,914 (70.5)	1,311 (57.7)	< 0.001
	-2 to -3SD	2,793 (13.6)	2,443 (13.3)	350 (15.4)	
	>-2SD	3,584 (17.4)	2,972 (16.2)	613 (27.0)	
	Missing	0	0	0	
Vaccination	Up to date	6,950 (97.4)	6,145 (97.5)	805 (96.9)	0.3
	Missing	13,468 (65.4)	12,025 (65.6)	1,443 (63.5)	
Wheeze	Present	3,240 (16.2)	2,874 (16.1)	805 (16.6)	0.54
	Missing	545 (2.7)	475 (2.6)	70 (3.1)	
Anaemia	Present	1,000 (4.9)	909 (5.0)	91 (4.0)	0.045
	Missing	0	0	0	
HIV	Positive or Exposed	231 (1.1)	197 (1.1)	34 (1.5)	0.073
	Missing	1	1	0	
TB	Positive	161 (0.8)	120 (0.7)	41 (1.8)	< 0.001
	Missing	0	0	0	
Neurological disorder	Present	22 (0.1)	12 (0.01)	10 (0.4)	< 0.001
	Missing	0	0	0	
Saturations < 90%		7,717 (37.5)	6,946 (37.9)	771 (33.9)	< 0.001

*WAZ* weight-for-age Z-score; Abnormal WAZ <-2SD

Neurological disorder: cerebral palsy, convulsive disorder or epilepsy

*P*-values by t-test for continuous variables and Chi^2^ for binary/categorical variables

### Factors associated with severe pneumonia re-admissions

Age (12–59 months vs. 2–11 months: adjusted odds ratio (aOR) 1.70, 95% 1.54 to 1.87), over 5 years vs. 2–11 months: aOR 1.85, 95% 1.55 to 2.22), severe malnutrition (WAZ <-3SD vs. WAZ> -2SD) (aOR 2.05, 95%1.84 to 2.29), moderate malnutrition (WAZ <-3SD vs. WAZ> -2SD) (aOR 1.37, 95%1.20 to 1.57), wheeze (aOR 1.17, 95% 1.03 to 1.33), and neurological disorder (aOR 4.42, 95% 1.70 to 11.48) were independent risk factors for re-admission adjusting for gender, vaccination status, any wheeze, anaemia, HIV disease and TB (Table 3). Older age was an independent risk factor for readmission in most one-year age windows when compared to age 2–11 months (Supplementary Table 1). Independent risk factors for re-admission among children aged 2–11 months were severe malnutrition, moderate malnutrition, and vaccination; and among 12–59 months were age 24–35 months, severe malnutrition, moderate malnutrition, and neurological disorder (Supplementary Tables 2, 3 and 4).

**Table 3:** Risk factors for readmission among study participants

Factor	Adjusted odds ratio (95% confidence interval)	*p*-value
Age (ref: <12 months)		
12–59 months	1.70 (1.54–1.87)	< 0.001
Over 5yrs	1.85 (1.55–2.22)	< 0.001
Male (ref: female)	1.04 (0.95–1.14)	0.391
WAZ (ref: >-2SD)		
-2 to -3SD	1.37 (1.20–1.57)	< 0.001
<-3SD	2.05 (1.84–2.29)	< 0.001
Vaccination (ref: incomplete)	0.71 (0.46–1.08)	0.093
vWheeze (ref: absent)	1.17 (1.03–1.33)	0.013
Clinical pallor (ref: absent)	0.97 (0.77–1.23)	0.790
HIV (ref: negative)	1.16 (0.78–1.74)	0.470
TB (ref: absent)	1.39 (0.94–2.04)	0.097
Neurological disorder (ref: absent)	4.42 (1.70-11.48)	0.002

Number of imputations=10; Number of observations=20,602; Number of groups=20; Outcome = severe pneumonia readmission

Imputed variables = gender 127; wheeze 545; vaccination 13,468

*WAZ* Weight-for-age Z-score

Neurological disorder: cerebral palsy, convulsive disorder or epilepsy

### Investigations and treatment provided to study participants

**Fig. 2:**
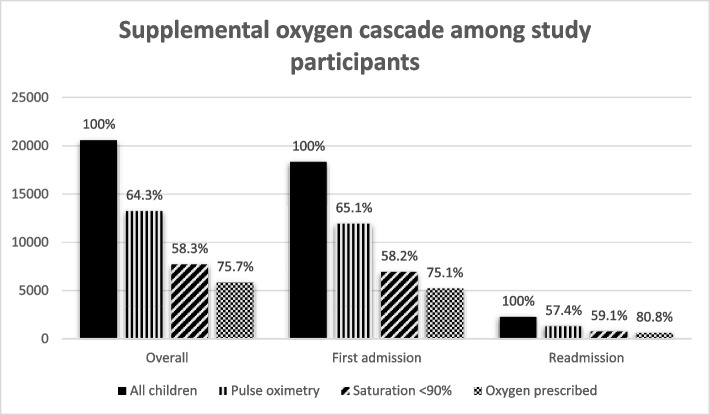
Supplemental oxygen cascade among study participants Percentages derive on denominators from the preceding bars

 Pulse oximetry was documented in 13,245/20,603 (64.3%) children. Children re-hospitalised with severe pneumonia compared to children admitted for the first time with severe pneumonia had less frequent documentation of pulse oximetry (1,305/2,274 [57.4%] vs. 11,940/18,329 [65.1%], *p* < 0.001). Oxygen prescription frequencies for children with severe pneumonia and documented saturations < 90% (hypoxia) in the overall group, first hospitalisation and readmission groups were 5,839/7,717 (75.7%), 5,216/6,946 (75.1%) and 623/771 (80.8%) respectively (Fig. 2). Compared to children admitted for the first time with severe pneumonia, children re-admitted with severe pneumonia had chest radiography performed more frequently (540/2,274 [23.7%] vs. 3,102/18,329 [16.9%], *p* < 0.001); and more prescriptions for second line antibiotics, specifically ceftriaxone (808/2,274 [35.5%] vs. 5,538/18,329 [30.2%], *p* < 0.001), TB treatment (69/2,274 [3.0%] vs. 298/18,329 [1.6%], *p* < 0.001), salbutamol (530/2,274 [23.3%] vs. 3,707/18,329 [20.2%], *p* = 0.003) and prednisone (157/2,274 [6.9%] vs. 764/18,329 [4.2%], *p* < 0.001) (Table 4).

**Table 4:** Diagnostics and treatments used among study participants

		Total(*N* = 20,603)	Severe Pneumonia First Admission (*N* = 18,329)	Severe Pneumonia Readmission (*N* = 2,274)	*p*-value
INVESTIGATIONS (%)					
Pulse oximetry done	Yes	13,245 (64.3)	11,940 (65.1)	1,305 (57.4)	< 0.001
	No	7,358 (35.7)	6,389 (34.9)	969 (42.7)	
Chest radiography	Yes	3,642 (17.7)	3,102 (16.9)	540 (23.8)	< 0.001
	No	16,961 (82.3)	15,227 (83.1)	1,734 (76.3)	
TREATMENTS (%)					
Oxygen ordered	Yes	5,839 (28.3)	5,216 (28.5)	623(27.4)	0.54
	No	14,764 (71.7)	13,113 (71.5)	1,651 (72.6)	
First line antibiotics					
Crystalline penicillin	Yes	16,376 (79.5)	14,721 (80.3)	1,655 (72.8)	< 0.001
	No	4,227 (20.5)	3,608 (19.7)	619 (27.2)	
Gentamicin	Yes	14,613 (70.9)	13,233 (72.2)	1,380 (60.7)	< 0.001
	No	5,990 (29.1)	5,096 (27.8)	894 (39.3)	
Amoxicillin	Yes	1,220 (5.9)	1,104 (6.0)	116 (5.1)	0.20
	No	19,383 (94.1)	17,225 (94.0)	2,158 (94.9)	
Second line antibiotics					
Ceftriaxone	Yes	6,346 (30.8)	5,538 (30.2)	808 (35.5)	< 0.001
	No	14,257 (69.2)	12,791 (69.8)	1,466 (64.5)	
Other antimicrobials					
Metronidazole	Yes	1,002 (4.9)	894 (4.9)	108 (4.7)	0.92
	No	19,601 (95.1)	17,435 (95.1)	2,166 (95.3)	
Cotrimoxazole	Yes	538 (2.6)	480 (2.6%)	58 (2.6)	0.94
	No	20,065 (97.4)	17,849 (97.4)	2,216 (97.4)	
TB medication	Yes	367 (1.8)	298 (1.6%)	69 (3.0)	< 0.001
	No	20,236 (98.2)	17,875 (98.4)	2,205 (97.0)	
Other treatment					
Salbutamol	Yes	4,237 (20.6)	3,707 (20.2)	530 (23.3)	0.003
	No	16,366 (79.4)	14,622 (79.8)	1,744 (76.7)	
Prednisolone	Yes	921 (4.5)	764 (4.2%)	157 (6.9)	< 0.001
	No	19,682 (95.5)	17,565 (95.8)	2,177 (93.1)	

### Outcomes of study participants

The median length of hospital stay was 4 days IQR [2–7] for children hospitalised with a severe pneumonia for the first time; and 5 days IQR [3–8] for children readmitted with severe pneumonia. We noted that children readmitted with severe pneumonia were hospitalised for longer than seven days more frequently than those admitted for the first time with severe pneumonia (34% vs. 26%, *p* < 0.001). Among 18,329 children admitted with severe pneumonia for the first time, 2,354 (12.8% [12.4–13.3]) died during their hospital stay, while 269 (11.8% [10.6–13.2]) of 2,274 children readmitted with severe pneumonia died during their hospital stay (Table 5).

**Table 5:** Outcomes of study participants

	Total(*N* = 20,603)	Severe Pneumonia First Admission (*N* = 18,329)	Severe Pneumonia Readmission (*N* = 2,274)	*p*-value
Length of stay				
Median [IQR] (Range) days	4 [2–7] (1,401)	4 [2–7] (1,339)	5 [3–8] (1,401)	
Missing	1	1	0	
Less than 7 days	15,159 (73.6)	13,650 (74.5)	1,509 (66.4)	< 0.001
7 days or more	5,444 (26.4)	4,679 (25.5)	765 (33.6)	
Missing	0	0	0	
Less than 14 days	19,499 (94.4)	17,363 (94.7)	2,086 (91.7)	< 0.001
14 days or more	1,154 (5.6)	966 (5.3)	188 (8.3)	
Missing	0	0	0	
Less than 30 days	20,437 (99.2)	18,189 (99.2)	2,248 (98.9)	0.056
30 days or more	166 (0.8)	140 (0.8)	26 (1.1)	
Missing	0	0	0	
Discharged	17,261 (83.8)	15,337 (83.7)	1,924 (84.6)	0.18
Referred	641 (3.1)	567 (3.1)	74 (3.3)	
Died	2,623 (12.7)	2,354 (12.8)	269 (11.8)	
Missing	78 (0.4)	71 (0.4)	7 (0.3)	

*P*-values by t-test for continuous variables and Chi^2^ for binary/categorical variables

### Risk factors associated with mortality

**Fig. 3:**
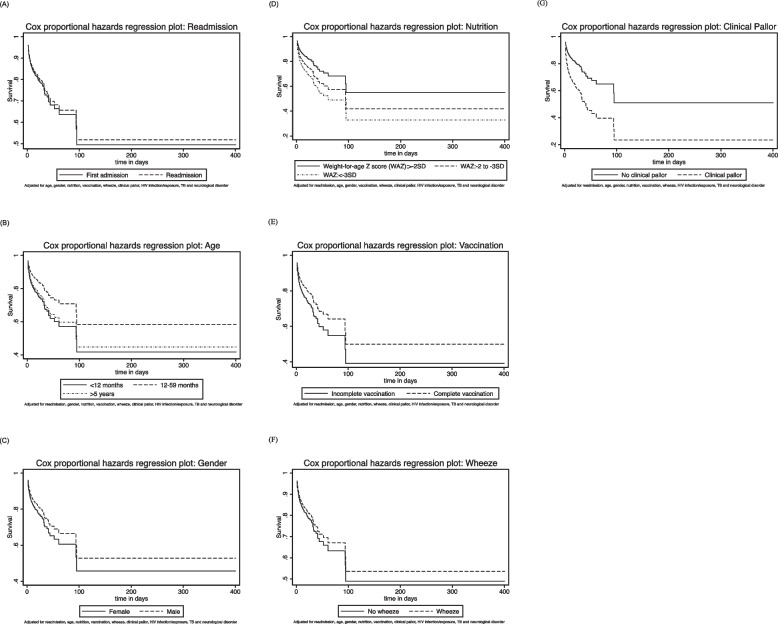
Cox proportional hazard plots for mortality among study participants

 Age 12–59 months (adjusted HR (aHR) 0.62, 95% CI 0.57 to 0.67), male gender (aHR 0.81, 95% CI 0.75 to 0.88), severe malnutrition (aHR 1.87 95% CI 1.71 to 2.05), moderate malnutrition (aHR 1.46, 95% CI 1.31 to 1.63), complete vaccination (aHR 0.74, 95% CI 0.60 to 0.91), wheeze (aHR 0.87, 95% CI 0.78 to 0.98) and clinical pallor (aHR 2.14, 95% CI 1.89 to 2.43) were independently associated with mortality amongst children admitted with severe pneumonia when adjusted for pneumonia readmission, HIV, TB and a neurological disorder (Table 6; Fig. 3).

**Table 6:** Factors associated with mortality among children admitted with severe pneumonia in Kenyan hospitals within the Clinical Information Network (CIN)

Factor	Adjusted hazard ratio (95% CI)	*p*-value
Pneumonia re-admission (ref: first episode pneumonia)	0.93 (0.82–1.07	0.312
Age (ref: <12 months)		
12–59 months	0.62 (0.57–0.67)	< 0.0001
Over 5yrs	0.92 (0.80–1.06)	0.254
Male (ref: female)	0.81 (0.75–0.88)	< 0.0001
WAZ (ref: >-2SD)		
-2 to -3SD	1.46 (1.31–1.63)	< 0.0001
<-3SD	1.87 (1.71–2.05)	< 0.0001
Vaccination up to date (ref: incomplete)	0.74 (0.60–0.91)	0.004
Wheeze present (ref: absent)	0.87 (0.78–0.98)	0.021
Clinical pallor present (ref: absent)	2.14 (1.89–2.43)	< 0.0001
HIV infected/exposed (ref: negative)	1.10 (0.84–1.45)	0.477
TB positive (ref: absent)	1.10 (0.75–1.60)	0.638
Neurological disorder (ref: absent)	1.23 (0.45–3.35)	0.682

Number of imputations=10; Number of observations=20,564; Number of groups=20; Outcome = severe pneumonia readmission

Imputed variables = gender 127; wheeze 545; vaccination 13,468; time 1102

*WAZ* weight-for-age Z-score

Neurological disorder: cerebral palsy, convulsive disorder

## Discussion

Our analysis revealed a high proportion of children aged 2 months to 14 years with severe pneumonia in Kenyan secondary-level health facilities had previously been admitted for care. Risk factors for re-hospitalisation for severe pneumonia are age > 12months, moderate/severe undernutrition, wheeze, and neurological disorder. The proportion of children getting plain chest radiography, second line antibiotics, TB medication, salbutamol and prednisone was modestly higher among those re-admitted compared to those hospitalised for the first time. Mortality among children hospitalised with severe pneumonia is high, but not significantly different among those readmitted compared to first admissions. Children who are < 12 months, over 5 years, female, with moderate/severe undernutrition clinical pallor or no wheeze are at a higher risk of dying.

Publications on severe pneumonia readmission in children in LMICs are limited. Conversely, financial incentives and penalties imposed by hospital readmission reduction programs seem to drive such studies in high income countries (HICs), more so among adults [28]. Re-admissions can be classified as early ($\le$30 days) or late ($\ge$31 days) [29]. Re-admissions that occur within 30-days often correspond to those that are potentially avoidable, most of which are direct or indirect complications of underlying comorbidities [30]. These are the readmissions health facilities and insurance companies are keen on reducing, and which hospitals in HICs are subject to penalties if unacceptable thresholds are reached [31]. Large longitudinal studies of various tertiary referral hospitals in the United States of America (USA), reported a 30-day lower respiratory infection specific re-admission rate and paediatric pneumonia specific re-admission rate of 2.7% [32] and 3.3% [5] respectively, almost four times lower than in our study. A more recent analysis conducted in the USA using the 2018 Nationwide Readmissions Database reported a 30-day severe pneumonia specific re-admission rate among children of 8.7%, while the re-admission rate in children with non-severe pneumonia was 4.7% [33]. A severe pneumonia readmission prevalence of 11% is higher than reports in the literature (2.7–8.7%) and is likely dependent on the context. In high income contexts such as the USA, patient level risk factors such as anaemia and malnutrition are much lower than those reported in our analysis. In addition, children in highly resourced settings may not experience system level barriers to health care such as those that may be more common in the African setting. Children in HICs have easier access to intensive outpatient facilities that are well equipped, sufficiently staffed and with adequate referral channels to tertiary/quarternary hospitals, where children with chronic conditions are likely to receive highly specialised treatment. The lower estimates found in the USA may also be attributed to pneumonia definitions, and the fact that these were strictly 30-day outcomes. All the children in our analysis had severe pneumonia based on WHO guidelines. Furthermore, we could only ascertain that they had a prior hospital admission, but we could neither determine the reason for the initial admission nor compute the time from the first admission given the nature of the data. It is imperative to obtain this time variable in future studies to report on comparable data regarding early and late pneumonia readmissions.

Hospital re-admission may signal failure in the health system performance. However, its interpretation may be complicated by observed and unobserved differences in the clinical risk of patient populations and their survival rates. For example, health facilities with low childhood mortality may have a higher unobserved volume of sick children at risk of re-admission [34]. In our study, mortality among children hospitalised with severe pneumonia was high, but not significantly different among those readmitted compared to first admissions. Indeed, hospital re-admissions are a more complex phenomenon than mortality, in that they arise from an “interplay among patient-, hospital-, community-, and environmental-level factors” [35]. Comorbidities including neurological disorders and malnutrition, were risk factors for re-hospitalisation for pneumonia in our study, findings similarly reported in the literature [5, 32]. In a cross-sectional study conducted at a Canadian tertiary hospital including almost 3,000 children, Owayed et al. reported that most children hospitalised with recurrent pneumonia have a predisposing factor, the most common being oropharyngeal incoordination [36]. This is a plausible explanation for the four-fold increased risk with severe pneumonia re-hospitalisation observed in children with a neurological disorder in our study. It calls for a high index of suspicion among clinicians from the first point of patient contact and consideration of medical or surgical interventions early. Immunosuppressive conditions have also been identified as a risk factor for pneumonia readmission in children [33]. Immunosuppression can be due to moderate/severe malnutrition which was significantly associated with readmission in this study. It is interesting that a higher proportion of children admitted for the first time with severe pneumonia were severely/moderately underweight compared to those readmitted. Notably, a higher proportion of children who were admitted for the first time with severe pneumonia were also younger, and perhaps more vulnerable to malnutrition. Moderate or severe malnutrition and neurological disorders in children with severe pneumonia should signal a need for post-admission follow up and potential re-admission. Optimizing nutrition may avert re-admission with severe pneumonia. Going forward, it will be important to conduct studies that objectively assess possible aspiration/reflux in children with neurological disorders readmitted with pneumonia in these settings perhaps using simple questionnaires at district-level health facilities [37] or/and more elaborate tests such as videofluoroscopic swallowing studies to assess swallowing [38] and scintigraphy, pH-metry and other studies to quantify gastro-oesophageal reflux in referral centres [39]. Establishing the multi-disciplinary capacity necessary to improve care for children with neurologic disorders, specifically training physiotherapists and speech therapists is an urgent requirement.

In this analysis, HIV was not associated with severe pneumonia readmission; perhaps due to timely initiation of antiretroviral therapy within prevention of mother-to-child transmission services [40]. Patient data on other conditions that present with recurrent pneumonia such as primary immunodeficiency, cystic fibrosis and primary ciliary dyskinesia that can lead to bronchiectasis [41] were not available. Unlike our study, age less than 1 year [5, 32] and male gender [32] have been identified as risk factors for re-hospitalisation for pneumonia. This could reflect differences in our study population or no actual gender risk for pneumonia rehospitalisation. There may be multiple factors that contribute to having a higher odds of readmission in older age groups compared with the 2–11-month age group. First, older children have more opportunity to be readmitted simply due to sequencing of events since readmission requires an initial admission at a younger age. Second, children may have respiratory related conditions that manifest later such as asthma, or pneumonia-related conditions/complications that develop later such as bronchiectasis. In both these cases, the exacerbations may become more apparent at an older age. Our additional analysis comparing narrower age windows (one-year intervals) was consistent with older age being an independent risk factor. Prior respiratory syncytial virus lower respiratory tract infection [42], longer index hospitalisations [5, 33], complicated pneumonia [5], and hospital case volume and teaching status [33] were other risk factors for re-hospitalisation for pneumonia identified in the literature but were not measured in this study. These are important variables that need to be examined in future studies.

Many of the children readmitted with severe pneumonia in our analysis were likely recurrent pneumonia cases based on a few plausible reasons. First, we could ascertain that they had at least two hospitalizations. Second, readmission was significantly associated with wheeze [43], malnutrition and neurological conditions [5, 32, 43], associated comorbidities that have been reported in the literature. Lastly, their median duration of hospitalisation was 5 days, with 99% of the children being hospitalized for < 30 days and 85% being discharged, signifying interval clinical improvement. This group of children with recurrent pneumonia is likely to be heterogenous [8]. Wheeze as a sign may be a proxy for asthma, as we note its positive association with readmission and negative association with mortality, suggesting a relatively less severe clinical phenotype. Baseer et al. estimated that 11.4% of children admitted with pneumonia during a one-year study in Egypt had recurrent pneumonia, a proportion similar to our study [43]. We further postulate that there may have been a smaller proportion of children with persistent pneumonia in both the first admission and readmission groups evidenced by a long hospital stay of ≥ 30 days serving as a proxy for persistent symptoms. In our analysis, almost 20% of children had a chest radiograph, with a significantly higher frequency of requests among children who were readmitted compared to those admitted with pneumonia for the first time. Data on interval assessment of chest radiographs was not available. It may be useful in future studies to examine chest radiography findings and ascertain if children readmitted with severe pneumonia have recurrent pneumonia/persistent pneumonia based on interval clearance or persistence of opacities respectively. Researchers in the future should aim to characterize children with recurrent/persistent pneumonia better as timely interventions could potentially reverse, prevent or better manage chronic lung disease [8, 44].

Similar to our findings, other studies conducted in comparable epidemiological and geographical contexts, specifically Kilifi at the Kenyan coast, Bangladesh and India, have found malnutrition [45–47] and incomplete vaccination [48] to be associated with mortality in children with severe pneumonia. We found clinically assessed pallor to be associated with mortality in children with severe pneumonia [47, 49], consistent with previous findings among children with non-severe pneumonia [6]. This association in children with pneumonia may be indicative of a reduced threshold for respiratory decompensation in this vulnerable population. Timely identification and management of anaemia is needed to prevent death in children hospitalised with severe pneumonia. Previous studies have described the association between higher mortality and infants with severe pneumonia [50], which we also found in our analysis. Interestingly, mortality in children over 5 years was similar to that observed among infants. Mortality among children aged 5–14 years has received little attention [51], with those in sub-Saharan Africa faring worse [52]. Older children may have attendant complex comorbidities that put them at risk for death. Evidence-based treatment algorithms for this neglected population are needed.

Approximately two thirds of children hospitalised with severe pneumonia in our analysis had documented pulse oximetry, an essential vital sign [53]. In an analysis of CIN hospitals between 2014 and 2020, Tuti et al. found that pulse oximetry was documented in only 49% of admission cases, with increasing but variable adoption in different hospitals [54]. Barriers to pulse oximetry adoption reported in the literature include lack of equipment, poor maintenance, inadequate local leadership, clinical training and pulse oximetry promotion in respective health facilities [54, 55]. Although our data suggests improvement in documented pulse oximetry use, 64% is sub-optimal. In addition to these barriers, it is possible that pulse oximetry was measured but not documented. Surprisingly, pulse oximetry was less frequently documented in children re-admitted with severe pneumonia compared to those admitted for the first time (57% vs. 65%) and could have underestimated hypoxia in this group of children. These crude associations are subject to confounding. Children who were readmitted may have been so severely ill that pulse oximetry was skipped or conversely, they may have displayed clinical characteristics that were less likely to prompt pulse oximetry assessment. Overall, oxygen was ordered for most of the children documented to be hypoxic (76%), with higher oxygen prescription frequencies among hypoxic children who were readmitted compared to those hospitalised for the first time (81% vs. 75%). This differentially lower documentation of pulse oximetry and higher oxygen prescription among hypoxic children readmitted with severe pneumonia compared to those admitted for the first time is interesting and may point to other factors that may influence management, including healthcare worker behavior regarding disease severity perception. Interrogating the supplemental oxygen cascade comprehensively and prospectively from measurement to appropriate action may be required. In addition to assessing documentation, we may need to use a robust mixed methods approach [55–57] that is contemporary, multidimensional and iterative. Important considerations include: (i) observing and enquiring about healthcare worker behavior; (ii) setting national/global targets for supplemental oxygen cascade documentation in admission records e.g. 95% for pulse oximetry documentation and 95% for correct oxygen supplementation in hypoxic patients; and (iii) providing timely solutions to barriers identified in the provision of pulse oximetry and appropriate oxygen supplementation.

In this study, referral of patients with severe pneumonia who had been readmitted was quite low at 3%, similar to those with a first hospitalisation with pneumonia. It is difficult to ascertain whether clinicians are reluctant to refer patients; or whether patients would honour the referral instructions. Exploring referral systems in future studies may prompt changes in patient pathways for children with severe pneumonia who have been re-hospitalised. This may inform the health facilities on the areas that could be improved, which could include increasing the number of specialists and sub-specialists in the counties through models such as the African Paediatric Fellowship Program [58]. Remote consultation [59], among other solutions that adopt a contextually appropriate reasoned diagnostic approach for children with recurrent/persistent pneumonia may be considered [8].

To the best of our knowledge, this is the first published study to report on severe pneumonia rehospitalisation in children in sub-Saharan Africa. The large sample size across multiple settings provides a contemporary representation of paediatric severe pneumonia re-admission in a high burden context, offering a foundation for research and interventions averting or mitigating the clinical sequelae of pneumonia. We used the WHO 2013 guidelines to classify severe pneumonia for all the children included in this analysis, therefore changes in national guidance did not affect our study findings. Similarly, antibiotic recommendations may not have affected our findings substantially since the population studied was hospitalized, with majority receiving intravenous antibiotics and only 6% receiving amoxicillin. The study is limited by its retrospective nature and use of routinely collected data, which may result in missing data on variables of interest, such as the interval between first admission and subsequent readmissions, challenges linking records for patients with multiple episodes of readmission, specialised modalities of testing employed (e.g., videofluoroscopy, echocardiography), relevant complications (e.g., pleural effusions, abscess, air leaks), and underestimating diagnostics and treatments received. Our results represent a conservative estimate of diagnostics and treatments available. Although the proportion of missingness of vaccination status is 65%, the proportion of missingness of other auxiliary variables in our multivariable models was minimal. Our multiple imputation approach employing 10 imputations in a large data set of > 20,000 participants under the assumption of MAR is robust in providing unbiased estimates with improved efficiency when compared to complete case analysis. There is limited evidence regarding missing data thresholds for which multiple imputation should not be performed [60, 61]. Previous analyses have examined the validity of the assumption of MAR using CIN data [62]. There is potential for misclassification of the vaccination status. If there was a systematic bias towards underreporting or overreporting vaccination status being up to date, then our analysis may have underestimated or overestimated the association respectively. It was not possible to reach patients and prospectively verify vaccination status using the mother child booklet. Nonetheless our results are consistent with what would be plausible and documented elsewhere in the literature.

## Conclusion

The proportion of children re-hospitalised with severe pneumonia and their in-hospital mortality is high. Although in-hospital mortality is similar in the re-admission group and first-time pneumonia admission group, 12.8% and 11.8% respectively are substantial proportions of death in children. Age, nutritional status, wheeze and neurological disorder are risk factors of severe pneumonia recurrence; additionally, gender, incomplete vaccination and clinical pallor, are associated with increased risk of mortality. Optimal care for severe pneumonia will require carefully designed studies of clinical algorithms for the screening, case management, and follow up of children, prioritising those with underlying risk factors.

### Supplementary Information

**Supplementary Material 1.**

## Data Availability

The data utilized in this work was made available to the research team by the participating hospitals and the Ministry of Health, and thus we are not the primary data owners; our use for these routine hospital data is approved as part of a specific ethical review process. Further access to the data can be sought through a request to KEMRI Wellcome Trust Research Programmes’s Data Governance Committee through email: dgc@kemri-wellcome.org.

## Abbreviations

aHR: Adjusted Hazard Ratio
aOR: Adjusted Odds Ratio
CIN: Clinical Information Network
KEMRI: Kenya Medical Research Institute
LMIC: Low-and-middle-income country
TB: Tuberculosis
WAZ: Weight-for-Age Z-score
WHO: World Health Organization
