# Supplementary material for: Characteristics of children readmitted with severe pneumonia in Kenyan hospitals
Source: BMC Public Health. 2024 May 16;24:1324. doi: 10.1186/s12889-024-18651-2 (PMC11097591; doi:10.1186/s12889-024-18651-2)
Supplement: Supplementary file 1 — Supplementary Material 1. [file 12889_2024_18651_MOESM1_ESM.docx]

Supplementary Table 1: Risk factors for readmission among study participants exploring age windows

| Factor | Adjusted odds ratio  (95% confidence interval) | p-value |
| --- | --- | --- |
| Age (ref: 2-11 months) |  |  |
| 1-2 years | 1.61 (1.44-1.80) | <0.0001 |
| 2-3 years | 1.93 (1.67-2.23) | <0.0001 |
| 3-4 years | 1.65 (1.37-2.00) | <0.0001 |
| 4-5 years | 1.78 (1.41-2.25) | <0.0001 |
| 5-6 years | 2.04 (1.49-2.79) | <0.0001 |
| 6-7 years | 1.56 (1.05-2.33) | 0.029 |
| 7-8 years | 1.44 (0.92-2.25) | 0.106 |
| 8-9 years | 1.53 (0.87-2.69) | 0.135 |
| 9-10 years | 2.00 (1.21-3.31) | 0.007 |
| 10-11 years | 1.59 (0.84-3.03) | 0.156 |
| 11-12 years | 1.45 (0.61-3.48) | 0.401 |
| 12-13 years | 4.28 (2.38-7.71) | <0.0001 |
| 13-14 years | 4.16 (1.50-11.56) | 0.006 |
| Male (ref: female) | 1.04 (0.95-1.14) | 0.391 |
| WAZ (ref: >-2SD) |  |  |
| -2 to -3SD | 1.39 (1.22-1.58) | <0.0001 |
| <-3SD | 2.09 (1.87-2.34) | <0.0001 |
| Vaccination (ref: incomplete) | 0.73 (0.49-1.10) | 0.128 |
| Wheeze (ref: absent) | 1.17 (1.03-1.33) | 0.015 |
| Clinical pallor (ref: absent) | 0.96 (0.76-1.22) | 0.763 |
| HIV (ref: negative) | 1.13 (0.76-1.69) | 0.550 |
| TB (ref: absent) | 1.36 (0.92-2.01) | 0.121 |
| Neurological disorder (ref: absent) | 4.61 (1.78-11.91) | 0.002 |

Number of imputations=10; Number of observations=20,602; Number of groups=20; Outcome = severe pneumonia readmission.

Imputed variables = gender 127; wheeze 545; vaccination 13,468.

*WAZ* weight-for-age Z-score

Supplementary Table 2: Risk factors for readmission among study participants (Age 2-12 months)

| Factor | Adjusted odds ratio  (95% confidence interval) | p-value |
| --- | --- | --- |
| Male (ref: female) | 1.01 (0.87-1.18) | 0.849 |
| WAZ (ref: >-2SD) |  |  |
| -2 to -3SD | 1.64 (1.33-2.02) | <0.001 |
| <-3SD | 2.81 (2.38-3.33) | <0.001 |
| Vaccination (ref: incomplete) | 0.59 (0.36-0.97) | 0.038 |
| Wheeze (ref: absent) | 1.16 (0.94-1.43) | 0.172 |
| Clinical pallor (ref: absent) | 1.15 (0.79-1.68) | 0.467 |
| HIV (ref: negative) | 1.01 (0.51-1.98) | 0.982 |
| TB (ref: absent) | 2.16 (1.15-4.03) | 0.016 |
| Neurological disorder (ref: absent) | 2.19 (0.42-11.32) | 0.351 |

Number of imputations=10; Number of observations=9,906; Number of groups=20; Outcome = severe pneumonia readmission. *WAZ* weight-for-age Z-score

Supplementary Table 3: Risk factors for readmission among study participants (Age 12-59 months)

| Factor | Adjusted odds ratio  (95% confidence interval) | p-value |
| --- | --- | --- |
| Age (ref: 12-23 months) |  |  |
| 24-35 months | 1.18 (1.01-1.37) | 0.033 |
| 36-47 months | 1.00 (0.82-1.21) | 0.968 |
| 48-60 months | 1.07 (0.85-1.36) | 0.548 |
| Male (ref: female) | 1.04 (0.92-1.18) | 0.553 |
| WAZ (ref: >-2SD) |  |  |
| -2 to -3SD | 1.26 (1.05-1.51) | 0.012 |
| <-3SD | 1.71 (1.45-2.01) | <0.0001 |
| Vaccination (ref: incomplete) | 0.82 (0.44-1.54) | 0.519 |
| Wheeze (ref: absent) | 1.15 (0.97-1.36) | 0.115 |
| Clinical pallor (ref: absent) | 0.80 (0.57-1.13) | 0.207 |
| HIV (ref: negative) | 1.23 (0.64-2.35) | 0.535 |
| TB (ref: absent) | 1.17(0.66-2.05) | 0.594 |
| Neurological disorder (ref: absent) | 11.96 (2.13-67.23) | 0.005 |

Number of imputations=10; Number of observations=9,287; Number of groups=20; Outcome = severe pneumonia readmission. *WAZ* weight-for-age Z-score

Supplementary Table 4: Risk factors for readmission among study participants (Age 5-14 years)

| Factor | Adjusted odds ratio  (95% confidence interval) | p-value |
| --- | --- | --- |
| Age (ref: 5-6 years) |  |  |
| 6-7 years | 0.80 (0.48-1.32) | 0.387 |
| 7-8 years | 0.75 (0.44-1.29) | 0.299 |
| 8-9 years | 0.80 (0.42-1.52) | 0.504 |
| 9-10 years | 1.08 (0.59-1.96) | 0.805 |
| 10-11 years | 0.86 (0.42-1.75) | 0.671 |
| 11-12 years | 0.63 (0.25-1.60) | 0.327 |
| 12-13 years | 2.02 (1.02-4.02) | 0.045 |
| 13-14 years | 1.97 (0.66-5.85) | 0.225 |
| Male (ref: female) | 1.22 (0.86-1.71) | 0.263 |
| WAZ (ref: >-2SD) |  |  |
| -2 to -3SD | 1.31 (0.75-2.27) | 0.341 |
| <-3SD | 1.38 (0.91-2.10) | 0.135 |
| Vaccination (ref: incomplete) | 1.46 (0.19-11.46) | 0.717 |
| Wheeze (ref: absent) | 1.27 (0.82-1.97) | 0.288 |
| Clinical pallor (ref: absent) | 1.03 (0.54-1.97) | 0.930 |
| HIV (ref: negative) | 1.40 (0.61-3.21) | 0.423 |
| TB (ref: absent) | 0.86 (0.31-2.47) | 0.785 |
| Neurological disorder (ref: absent) | 7.76 (0.99-60.80) | 0.051 |

Number of imputations=10; Number of observations=1,409; Number of groups=20; Outcome = severe pneumonia readmission. *WAZ* weight-for-age Z-score
